# Intermittent Fasting Protects Against the Progression from Acute Kidney Injury to Chronic Kidney Disease

**DOI:** 10.3390/antiox14010119

**Published:** 2025-01-20

**Authors:** Yoonjoo Jang, Young Suk Kim, Seo Rin Kim, Dong Won Lee, Soo Bong Lee, Il Young Kim

**Affiliations:** 1 Department of Internal Medicine, Pusan National University School of Medicine, Yangsan 50612, Republic of Koreakim.seorin@pusan.ac.kr (S.R.K.);; 2 Research Institute for Convergence of Biomedical Science and Technology, Pusan National University Yangsan Hospital, Yangsan 50612, Republic of Korea

**Keywords:** acute kidney injury, chronic kidney disease, epithelial–mesenchymal transition, intermittent fasting, tubulointerstitial fibrosis

## Abstract

Acute kidney injury (AKI) is a major but often underestimated risk factor for the development of chronic kidney disease (CKD). Exploring innovative approaches to prevent this progression is critical. Intermittent fasting (IF), recognized for its metabolic and anti-inflammatory benefits, may offer protective effects in this context. Using a unilateral ischemia-reperfusion injury (UIRI) model in male C57BL/6 mice, we evaluated the impact of IF on tubulointerstitial fibrosis and tubular epithelial–mesenchymal transition (EMT) over 8 weeks. Mice in the IF group followed a 5:2 regimen, fasting for 24 h twice weekly. Four groups were studied: control, IF, UIRI, and IF + UIRI. The UIRI group exhibited increased fibrosis and EMT, both of which were significantly attenuated in the IF + UIRI group. IF also reduced levels of TGF-β1, phosphorylated NF-κB p65, inflammatory cytokines, and F4/80-positive macrophages, along with markers of oxidative stress. These findings highlight IF’s ability to mitigate fibrosis and EMT through reductions in inflammation and oxidative stress during AKI-to-CKD progression. Our study suggests that IF may serve as a promising dietary strategy to prevent AKI from advancing into CKD.

## 1. Introduction

Acute kidney injury (AKI) denotes a medical condition characterized by a swift deterioration in kidney function, often associated with significant morbidity and mortality [1,2]. Historically, AKI was considered a reversible condition. However, recent studies suggest that kidney recovery following AKI is often incomplete, with persistent structural impairment that may result in the development of chronic kidney disease (CKD) [2]. Both epidemiological and experimental research shows that the intensity and length of AKI episodes are predictive of future CKD development [3]. As a result, the progression from AKI to CKD has emerged as a central focus of kidney disease research. Unfortunately, there are currently no treatments that can successfully halt the advancement of AKI to CKD.

The progression from AKI to CKD involves a complex and not fully understood pathophysiology [4]. However, increasing evidence highlights the roles of tubular epithelial cell (TEC) damage, interstitial inflammation, fibrosis, and endothelial dysfunction as key contributors to this process [4]. Various factors, including ischemia, sepsis, inflammation, and exposure to nephrotoxic agents, can trigger AKI [5]. When compensatory mechanisms fail to repair the damage, the healing process becomes impaired (maladaptive repair), leading to defective angiogenesis, cell cycle disruption, epithelial–mesenchymal transition (EMT) in TECs, interstitial tissue expansion, and inflammatory cell infiltration in the injured kidney [6,7]. TECs are particularly crucial in the progression from AKI to CKD, responding to injury by secreting inflammatory mediators and signaling molecules that drive tissue inflammation and fibrosis in the interstitium. Moreover, TECs are thought to drive this transition through EMT [6,8]. Thus, TECs are not passive players but actively contribute to inflammation and fibrosis during the AKI-to-CKD progression.

Caloric restriction involves reducing daily caloric intake over an extended period. Studies in animals have shown that caloric restriction can improve health, extend lifespan, and reduce mortality rates [9]. However, caloric restriction is difficult to maintain and may increase the risk of malnutrition. Consequently, intermittent fasting (IF) has gained popularity as a more practical alternative and has received growing attention in recent years. IF consists of cycles of consumption and abstention from food (or significantly reduced calorie intake) and has become a widely adopted lifestyle approach [10]. Both animal and human studies have demonstrated the broad health benefits of IF, particularly in managing metabolic disorders, through mechanisms beyond simple caloric restriction [10]. Several clinical trials have indicated that IF may provide similar or even greater improvements in weight loss and insulin resistance compared to continuous caloric restriction, with better adherence to the regimen [11]. While preclinical studies consistently show that IF has strong disease-modifying effects across a range of persistent health issues, such as excessive weight gain, insulin resistance, heart-related disorders, malignancies, and neurodegenerative diseases [10], its impact on kidney health has been less thoroughly investigated. In this study, we explore whether IF can protect against kidney injury in a mouse model of AKI-to-CKD progression.

## 2. Materials and Methods

### 2.1. Animal Experimental Design

All procedures involving animals were conducted with approval from the Institutional Animal Care and Use Committee of Pusan National University (PNU-IACUC, L2023-023-A1C0) and in compliance with the standards set by the Korean Association for Laboratory Animals. Eight-week-old male C57BL/6 mice were obtained from Koatech Technology Corporation (Seoul, Republic of Korea) and were kept in an animal housing unit with a 12 h light/dark schedule at 22 °C, where they had unrestricted availability of food and water.

To create a murine model for the progression from AKI to CKD, UIRI was induced in mice without performing nephrectomy on the opposite side, following established protocols [12]. Briefly, the mice were anesthetized with isoflurane vapors, and an incision was made on the right flank to expose the right renal pedicle. Ischemia was induced by placing a microaneurysm clamp on the renal pedicle for 30 min, as indicated by a visible change in color from red to dark blue. Afterward, the clamp was released, and normal blood flow was confirmed by the restoration of the typical coloration. The kidney was repositioned, and the surgical site was sutured in two layers. In the sham surgery group, the same steps were followed, except the renal pedicle was left unclamped.

**Figure 1:**
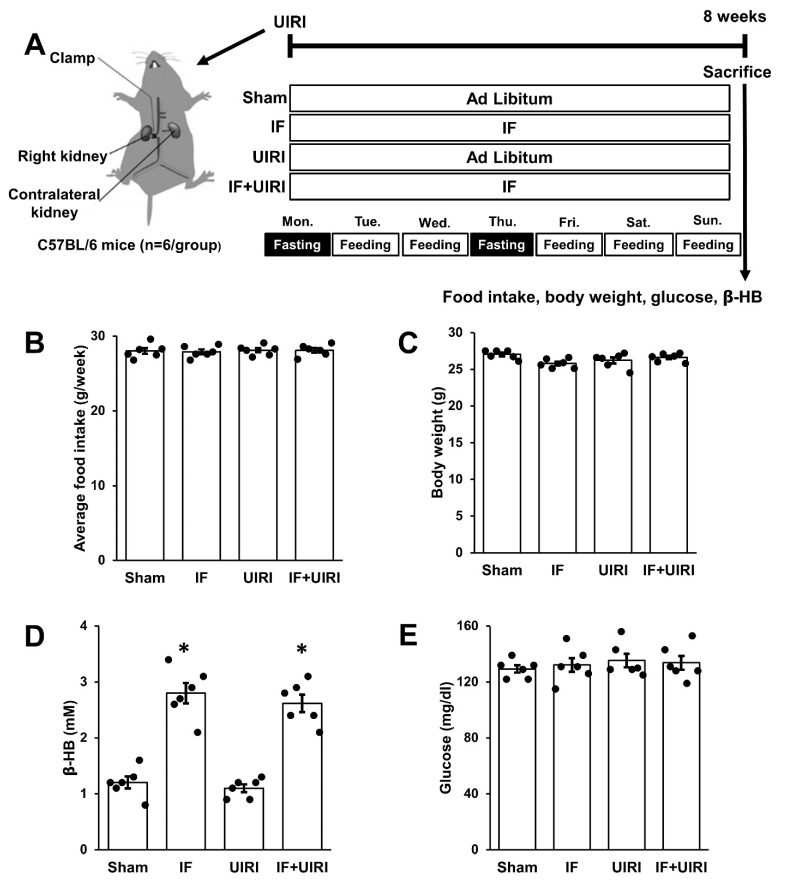
Effect of IF on metabolism. (**A**) Study setup: The ad libitum group had unrestricted food access throughout the day, whereas the IF group adhered to a 5:2 fasting schedule. In this regimen, the IF group fasted for a full day on two separate days each week, specifically on Monday and Thursday, from 9 a.m. to 9 a.m. (**B**) Mice subjected to IF for two months consumed food quantities comparable to those of the ad libitum group. (**C**) Body weight remained stable in the IF group. (**D**) The IF groups showed a marked increase in β-HB levels. (**E**) Blood glucose levels were similar between the IF and ad libitum groups. The measurements are shown as means ± SD (*n* = 6 per group; * *p* < 0.05 in comparison with sham and UIRI groups).

Following UIRI or sham surgeries, the mice were allocated into four groups (*n* = 6 per group) in a random manner (Figure 1A): (a) sham: free access to food without UIRI, (b) IF: IF without UIRI, (c) UIRI: free access to food with UIRI, and (d) IF + UIRI: IF with UIRI. Mice in the ad libitum groups were provided with unlimited access to food at all times, while those in the IF group followed a 5:2 diet regimen. The IF group and IF + UIRI group fasted for two non-consecutive days per week (Monday and Thursday) for 24 h each time (from 9 a.m. to 9 a.m.). Fasting for the IF and IF + UIRI groups commenced immediately after IRI or sham surgery on Monday, aligning with the start of the 5:2 IF regimen. After 8 weeks of either IF or ad libitum feeding, the mice were euthanized. Blood β-hydroxybutyrate (β-HB) and glucose levels were measured from blood samples obtained from the tail vein of fasted mice, using a Free-Style Optium Neo device (Abbott, CN, USA) after 8 weeks of either ad libitum feeding or IF. Furthermore, body weight and food intake were monitored and recorded on a weekly basis.

### 2.2. Histologic Examination

Paraffin-embedded kidney tissues were sliced into 3 μm thick sections for light microscopy analysis. As previously described [12], periodic acid-Schiff (PAS) and Masson’s trichrome (MT) staining were employed to assess tubular injury and interstitial fibrosis, respectively. Stained tissue slides were visualized using a ZEISS Axioscan7 (Carl Zeiss, Jena, Germany) and images were captured with ZEN Lite software version 3.9 (Carl Zeiss) at a magnification of 20×. Tubular damage was assessed by an evaluator unaware of the experimental groups, who analyzed 10 high-power fields (HPFs) of the renal cortex from PAS-stained samples, scoring based on the following scale: 0 for no damage, 1 for less than 10% damage, 2 for 10–25%, 3 for 25–50%, 4 for 50–75%, and 5 for more than 75%. Tubular damage was identified by various signs, such as thickening of the tubular basement membrane, cast formation, shedding of tubular epithelial cells (TECs), loss of the brush border, dilation, and atrophy. For interstitial fibrosis, MT-stained sections were examined in 10 cortical high-power fields, with scoring based on the following criteria: 0 for no fibrosis, 1 for fibrosis affecting up to 25%, 2 for fibrosis involving 25–50%, and 3 for fibrosis affecting more than 50% [12].

### 2.3. Immunohistochemical Analysis

Immunohistochemical analysis was performed on 3 μm thick paraffin-embedded tissue slices. Following established protocols [13], the sections were first deparaffinized using xylene and gradually rehydrated through a series of ethanol solutions. Antigen retrieval was accomplished by heating in a microwave. Non-specific binding was blocked using normal horse serum (Vector Laboratories, CA, USA), after which the tissue samples were placed at 4 °C overnight and exposed to primary antibodies. Afterward, secondary antibodies (ImmPRESS™ HRP reagent kit, Vector Laboratories) were applied at 37 °C for 30 min. The immunoreaction was visualized using DAB, followed by counterstaining with hematoxylin. The primary antibodies included anti-F4/80 (Abcam, Cambridge, UK), anti-E-cadherin (Cell Signaling Technology, MA, USA), anti-TGF-β1 (Abcam), anti-8-OHdG (JalCA, Sizuoka, Japan), anti-vimentin (Abcam), anti-nitrotyrosine (Abcam), anti-phosphorylated NF-κB p65 (Santa Cruz, CA, USA), anti-4-HNE (JalCA), and anti-α-smooth muscle actin (SMA) (Abcam). Images of the stained slides were captured with a ZEISS Axioscan 7 and analyzed using ZEN Lite software. Ten high-power fields (HPFs) were randomly chosen for evaluation. Quantification of 8-OHdG, p-NF-κB p65, and F4/80-positive cells was performed, and semi-quantitative scoring for 4-HNE, vimentin, α-SMA, TGF-β1, E-cadherin, and nitrotyrosine was carried out using the following system: 0 for no staining, 1 for staining covering 1–25% of the area, 2 for staining covering 26–50% of the area, 3 for staining covering 51–75% of the area, and 4 staining covering more than 75% of the area [13].

### 2.4. Quantitative Real-Time Polymerase Chain Reaction (PCR) Analysis

Real-time PCR was conducted as outlined in previous studies [13], utilizing *GAPDH* as a reference gene along with target genes *IL-1β*, *TNF-α*, and *IL-6*. The amplification products were validated through a melting curve analysis, with temperature cycling at 95 °C for 15 s, then 60 °C for 15 s, followed by a concluding phase at 95 °C for 15 s. The relative gene expression changes were calculated using the 2^−ΔΔCT^ formula. The sequences of primers are listed in Table 1.

### 2.5. Statistical Analysis

All statistical evaluations were performed using SPSS version 27 (SPSS, Inc., Chicago, IL, USA). The information was assessed using the Mann–Whitney U test or Kruskal–Wallis test, depending on the circumstances. A *p*-value below 0.05 was considered to indicate a significant result. The measurements are shown as mean ± standard deviation (SD).

## 3. Results

### 3.1. Effect of IF on Metabolism

Over the course of two months, the IF group consumed a quantity of food similar to that of the ad libitum control group, confirming that IF did not involve caloric restriction during the feeding periods (Figure 1B). Importantly, no changes in body weight were observed in the IF group (Figure 1C). However, the β-HB levels were elevated in the IF group, indicating a state of ketosis induced by the fasting regimen (Figure 1D). Blood glucose levels remained comparable between the IF and ad libitum control groups (Figure 1E).

### 3.2. Effect of IF on Kidney Pathology and Function During the Progression from AKI to CKD

**Figure 2:**
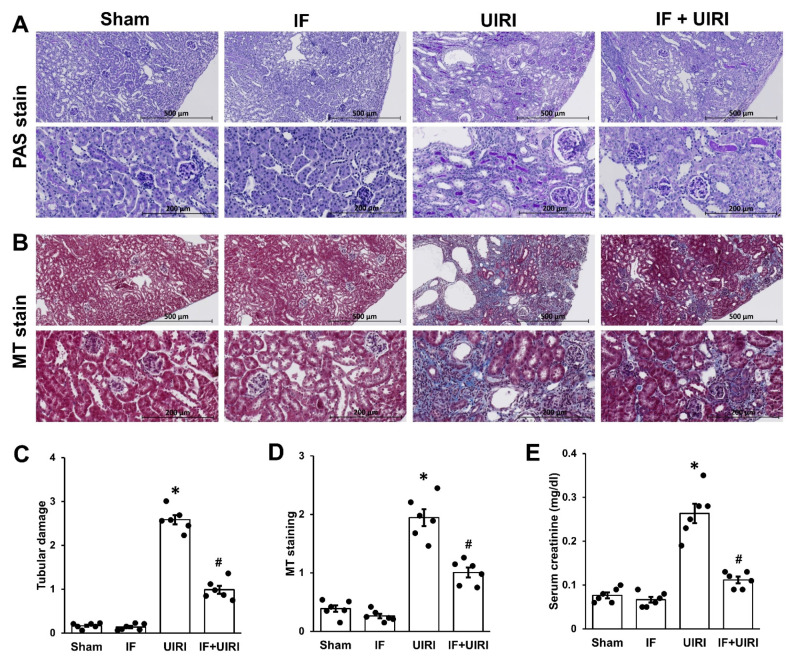
Effect of IF on kidney pathology and function during AKI-to-CKD progression. (**A**,**C**) Representative images stained with periodic acid-Schiff (PAS) reveal reduced tubular damage in the IF + UIRI group when contrasted with the UIRI group. (**B**,**D**) Masson’s trichrome (MT) staining reveals significantly reduced tubulointerstitial fibrosis in the IF + UIRI group relative to the UIRI group. (**E**) The IF + UIRI group showed reduced serum creatinine concentrations in comparison to the UIRI group. The measurements are shown as means ± SD (*n* = 6 per group; * *p* < 0.05 vs. sham and IF groups; # *p* < 0.05 vs. UIRI group).

Two months after UIRI, PAS staining showed significant tubular damage in the UIRI group, with noticeable dilation, atrophy, and cast formation compared to the sham group. These pathological alterations showed a marked reduction in the IF + UIRI group (Figure 2A,C). Similarly, MT staining revealed a marked increase in interstitial fibrosis in the UIRI group, whereas the IF + UIRI group exhibited a substantial reduction in fibrosis (Figure 2B,D). In line with these histological results, blood creatinine concentrations were higher in the UIRI group compared to the sham group, while the IF + UIRI group showed lower levels, suggesting that IF offers a protective effect on renal function during the progression from AKI to CKD (Figure 2E).

### 3.3. Effect of IF on Tubular EMT During the Progression from AKI to CKD

**Figure 3:**
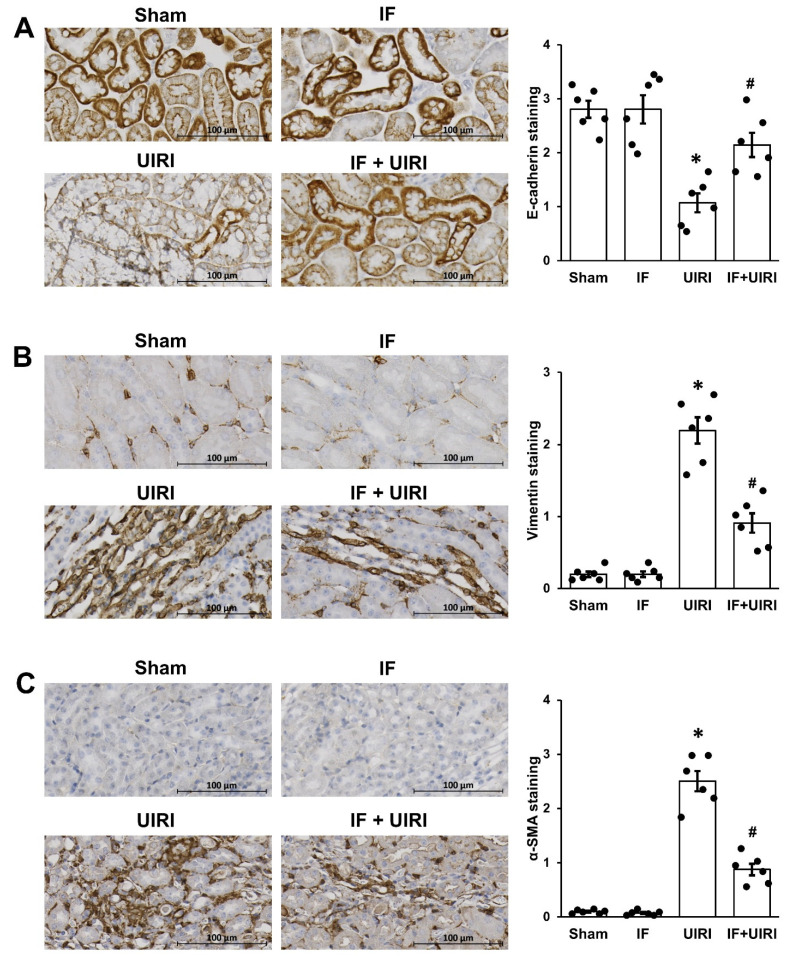
Effect of IF on tubular EMT during AKI-to-CKD progression. (**A**) Immunohistochemical analysis of E-cadherin revealed greater expression in the IF + UIRI group than in the UIRI group. (**B**,**C**) Immunohistochemical analysis of vimentin and α-SMA revealed reduced expression in the IF + UIRI group versus the UIRI group. The measurements are shown as means ± SD (*n* = 6 per group; * *p* < 0.05 vs. sham and IF groups; # *p* < 0.05 vs. UIRI group).

EMT in TECs is a known contributor to renal tubulointerstitial fibrosis [14]. Immunohistochemical analysis revealed a significant decrease in E-cadherin expression, an epithelial marker, in the UIRI group in relation to the sham group. However, this reduction was alleviated in the IF + UIRI group (Figure 3A). In contrast, the expression of mesenchymal markers, including α-SMA and vimentin, was elevated in the UIRI group in comparison with the sham group. These markers, however, showed a significant reduction in the IF + UIRI group relative to the UIRI group (Figure 3B,C).

### 3.4. Effect of IF on Inflammation During the Progression from AKI to CKD

**Figure 4:**
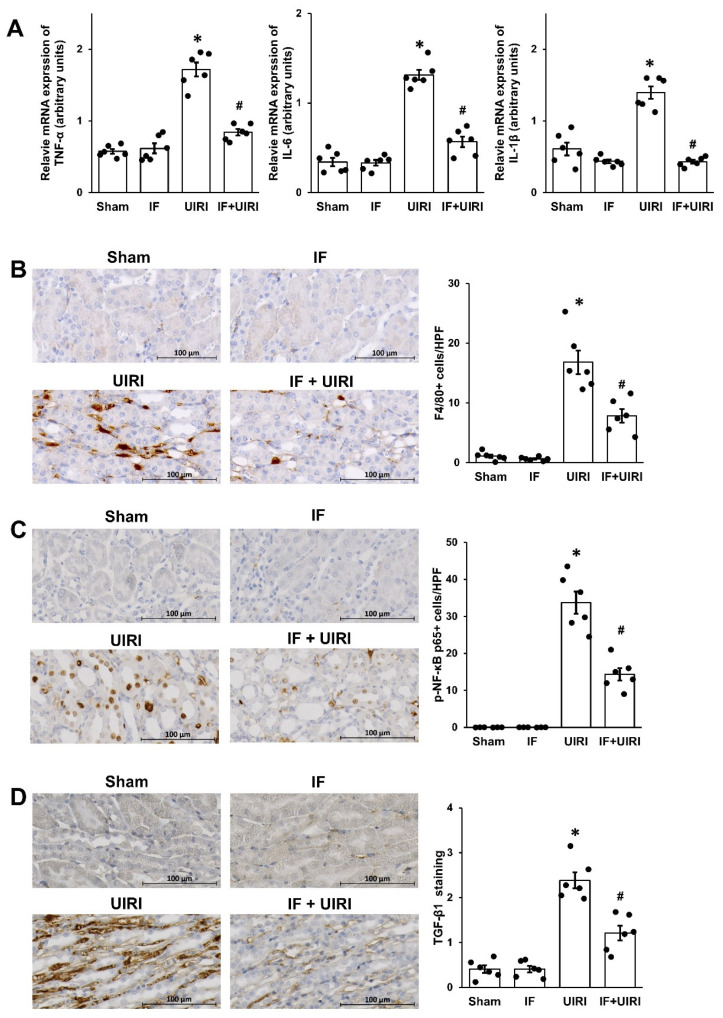
Effect of IF on inflammation during AKI-to-CKD progression. (**A**) mRNA expression of *TNF-α*, *IL-6*, and *IL-1β* was notably reduced in the IF + UIRI group relative to the UIRI group. (**B**) Immunohistochemical analysis revealed a decrease in F4/80-positive staining in the IF + UIRI group when assessed against the UIRI group. (**C**,**D**) Immunostaining demonstrated a reduction in phosphorylated NF-κB p65 and TGF-β1 levels in the IF + UIRI group, as compared to the UIRI group. The measurements are shown as means ± SD (*n* = 6 per group; * *p* < 0.05 vs. sham and IF groups; # *p* < 0.05 vs. UIRI group).

Inflammation is a key factor in tubular EMT and the development of kidney fibrosis [14]. Analysis of mRNA levels revealed that inflammatory mediators, such as *IL-6*, *IL-1β*, and *TNF-α*, were notably elevated in the UIRI group in contrast to the sham group. This increase was substantially reduced in the IF + UIRI group (Figure 4A). Additionally, the number of macrophages, identified by F4/80 staining in the interstitial region, was higher in the UIRI group than in the sham group, but this was notably reduced in the IF + UIRI group (Figure 4B). Considering the crucial roles of TGF-β and NF-κB in inflammation regulation [14], their expression levels were also examined. Immunohistochemical analysis revealed enhanced TGF-β1 and phosphorylated NF-κB p65 expression in the UIRI group, whereas these markers were considerably reduced in the IF + UIRI group (Figure 4C,D).

### 3.5. Effect of IF on Oxidative Stress During the Progression from AKI to CKD

**Figure 5:**
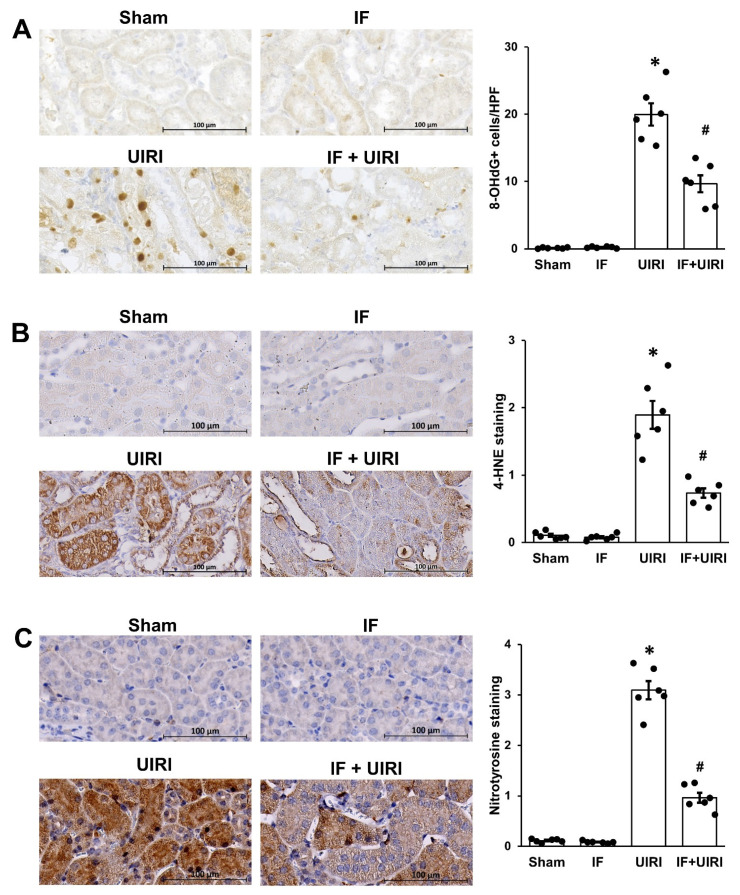
Effect of IF on oxidative stress during AKI-to-CKD progression. Immunohistochemical analysis revealed a decrease in oxidative stress markers, such as 8-OHdG (**A**), 4-HNE (**B**), and nitrotyrosine (**C**), in the IF + UIRI group in contrast to the UIRI group. The measurements are shown as means ± SD (*n* = 6 per group; * *p* < 0.05 vs. sham and IF groups; # *p* < 0.05 vs. UIRI group).

Oxidative stress is a key driver of tubular EMT and renal fibrosis [14]. To assess the impact of IF, kidney tissues were stained for markers of oxidative damage: nitrotyrosine (protein), 4-HNE (lipid), and 8-OHdG (DNA). The levels of all three markers were elevated in the UIRI group, while these markers of oxidative damage were reduced in the IF + UIRI group, suggesting that IF helps alleviate oxidative stress during the progression from AKI to CKD (Figure 5A–C).

## 4. Discussion

In our study, we used the UIRI model without contralateral nephrectomy, which is widely accepted for investigating the progression from AKI to CKD [15]. Renal IRI is a key contributor to AKI in humans, triggering a pathological cascade that results in acute tubulointerstitial damage [16]. Following IRI, a repair mechanism is activated to restore the kidney’s normal structure and function. However, in cases of severe injury, this can result in irreversible damage and progressive fibrosis [16]. Renal IRI is thus an important and valuable model for studying the transition from AKI to CKD. In our study, a mouse model of AKI-to-CKD progression was established, as evidenced by elevated serum creatinine levels and the onset of tubulointerstitial fibrosis. Notably, there was no significant difference in the degree of fibrosis in the left kidney (the kidney without IRI) among the four groups (MT staining score: sham, 0.21 ± 0.04; IF, 0.14 ± 0.02; UIRI, 0.13 ± 0.02; IF + UIRI, 0.13 ± 0.02, *p* = 0.302). The observed increase in serum creatinine in UIRI, despite the absence of contralateral nephrectomy, is thought to be due to the left kidney’s inability to sufficiently compensate for overall renal function 8 weeks after IRI of the right kidney. In this model, IF was shown to enhance renal function, reduce tubulointerstitial fibrosis and EMT, and decrease inflammation and oxidative stress in this model. As far as we are aware, this investigation offers the first evidence demonstrating the positive effects of IF on the transition from AKI to CKD.

Numerous investigations have examined the potential advantages of IF for maintaining health and addressing diseases [10]. Some studies connect the positive outcomes of IF to reduced energy consumption, while others emphasize enhanced metabolic indicators and resistance to adverse conditions, even with comparable calorie intake [17]. In this research, mice in the IF groups showed weight patterns and food intake similar to those of the control groups throughout the observation period, ruling out energy limitation. These findings imply that the benefits of IF on the transition from AKI to CKD observed in this study were not dependent on lower calorie consumption.

Several previous studies have investigated the effects of dietary interventions on the progression of AKI to CKD. Rojas-Morales et al. conducted a study in which they induced fasting for 3 days prior to kidney IRI and evaluated the degree of kidney fibrosis 14 days later. They concluded that fasting for 3 days before IRI suppressed kidney fibrosis, primarily due to the inhibition of oxidative stress and mitochondrial dysfunction. Another study by the same researchers demonstrated that fasting for 3 days prior to RI improved kidney fibrosis 28 days post-IRI, and they suggested that this effect was related to reductions in inflammation and endoplasmic reticulum stress, in addition to oxidative stress and mitochondrial dysfunction.

Several previous studies have investigated the effects of dietary interventions on the progression of AKI to CKD. Rojas-Morales et al. conducted a study in which they induced fasting for 3 days prior to kidney IRI and evaluated the degree of kidney fibrosis 14 days later [18]. They concluded that fasting for 3 days before IRI suppressed kidney fibrosis, primarily due to the inhibition of oxidative stress and mitochondrial dysfunction. Another study by the same researchers demonstrated that fasting for 3 days prior to IRI improved kidney fibrosis 28 days post-IRI, and they suggested that this effect was related to reductions in inflammation and endoplasmic reticulum stress, in addition to oxidative stress and mitochondrial dysfunction [19]. Our current study has several differences compared to the two studies by Rojas-Morales et al. First, we investigated the effect of IF (5:2 regimen) initiated after IRI on the progression of AKI to CKD, whereas the studies by Rojas-Morales et al. implemented extreme fasting (complete fasting for 3 days) before IRI. This indicates differences not only in the type of dietary intervention regimens but also in their application timing before and after IRI. Notably, 3 days of extreme fasting can cause significant stress to both mice and humans, making it difficult to apply in clinical settings. In contrast, the 5:2 IF regimen is already in practical use and holds potential for application to AKI-to-CKD progression in humans. Second, while the two studies by Rojas-Morales et al. examined kidney fibrosis at 2 and 4 weeks post-IRI, our study extended the evaluation period to 8 weeks. This difference allowed us to study kidney fibrosis at a more advanced stage. Third, the studies by Morales et al. revealed that fasting suppressed kidney fibrosis after IRI by reducing oxidative stress, mitochondrial dysfunction, inflammation, and endoplasmic reticulum stress. In contrast, our study demonstrated that intermittent fasting not only reduces inflammation and oxidative stress but also suppresses EMT, highlighting a key difference in findings.

A significant finding from our research is the marked reduction in inflammation induced by IF in the AKI-to-CKD progression. Although this investigation did not fully clarify the processes responsible for IF’s ability to reduce inflammation in this context, IF is widely recognized for its capacity to dampen inflammatory responses. Research in both human and animal models has shown that IF can alleviate inflammation in a variety of chronic inflammatory conditions, such as heart disease, metabolic disorders, neurological diseases, joint diseases, and different types of malignancies [20]. While these findings are compelling, the exact way through which IF reduces inflammation in the context of our investigation is not yet fully understood. One possible mechanism involves ketosis induced by IF. Higher levels of β-HB in mice undergoing IF suggest that this approach may lead to a state of ketosis. As a byproduct of liver fat metabolism, β-HB provides a substitute energy source when carbohydrates are scarce [21]. Beyond its metabolic role, β-HB plays an important role as a signaling molecule with the ability to influence inflammation by regulating forkhead box O1 (FOXO1) and peroxisome proliferator-activated receptor-gamma coactivator-1 alpha (PGC-1α) [21,22]. Further investigation is necessary to uncover the complex mechanisms through which IF impacts inflammation during the progression from AKI to CKD.

Additionally, our investigation has demonstrated that IF can decrease oxidative damage in a murine model of AKI-to-CKD progression. While we have not completely determined the precise mechanisms through which IF mitigates this transition, accumulating evidence points to IF’s potential to attenuate oxidative damage. A comprehensive review of controlled clinical trials indicates that IF may reduce markers of oxidative harm in individuals with obesity [23]. Previous studies in both animal and human models have indicated that IF can lower indicators of oxidative damage in a variety of chronic disorders [20]. Although the exact biochemical mechanisms remain partially unknown, it is probable that PGC-1α and nuclear factor erythroid 2-related factor 2 (NRF2)—transcription factors sensitive to redox changes—contribute to IF’s antioxidative properties [24]. Additionally, β-HB produced by IF has been shown to stimulate both FOXO1 and NRF2, which control genes that protect cells during the oxidative damage [25]. Further research is needed to uncover the intricate mechanisms through which IF influences oxidative stress in the transition from AKI to CKD.

Finally, our research has shown that IF reduces tubulointerstitial fibrosis and tubular EMT in a murine model of the progression from AKI to CKD. Tubulointerstitial fibrosis represents a crucial stage in the transition from AKI to CKD, marked by the stimulation of myofibroblasts, which are the key cells involved in the accumulation of interstitial extracellular matrix [26]. Although renal interstitial fibroblasts were previously thought to be the sole origin of myofibroblasts, increasing evidence indicates that injured TECs can undergo dedifferentiation, becoming a significant source of myofibroblasts [27]. Tubular EMT involves injured TECs losing epithelial markers and gaining mesenchymal characteristics, and it is recognized as a key mechanism underlying renal tubulointerstitial fibrosis [6]. In our study, the precise mechanism through which IF reduces tubulointerstitial fibrosis and tubular EMT remains unclear. However, accumulating evidence suggests that inflammation and oxidative stress work in tandem to induce EMT within the context of renal tubulointerstitial fibrosis. Notably, during inflammation associated with this fibrosis, TGF-β is recognized as the main factor driving tubular EMT, with NF-κB—activated by TGF-β—also playing a contributory role [14]. Under oxidative damage, reactive oxygen species are likewise implicated in tubular EMT [14]. As discussed, our findings show that IF mitigated both inflammation and oxidative stress throughout the transition from AKI to CKD. Given the well-established interactions between inflammation, oxidative stress, and tubular EMT, we propose that IF may reduce tubulointerstitial fibrosis and tubular EMT by alleviating inflammation and oxidative stress damage in the context of AKI-to-CKD progression.

## 5. Conclusions

**Figure 6:**
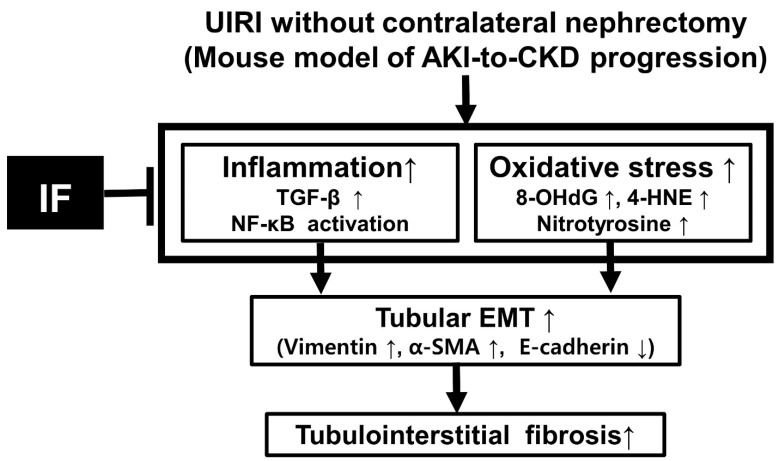
Summary of the protective effects of IF against tubulointerstitial fibrosis and tubular EMT in UIRI. This schematic highlights IF’s ability to alleviate tubulointerstitial fibrosis and tubular EMT by suppressing oxidative damage and inflammation in a murine model of AKI-to-CKD progression.

The outcomes of this research are summarized in Figure 6. An increase in tubulointerstitial fibrosis and tubular EMT was observed as a result of enhanced inflammation and oxidative damage in a murine model of AKI-to-CKD progression. IF was shown to alleviate tubulointerstitial fibrosis following tubular EMT. This protective effect of IF seemed to be associated with decreased inflammation and oxidative damage. As a result, IF could represent a novel dietary strategy to prevent the progression from AKI to CKD. Further investigations are necessary to clarify the mechanisms through which IF exerts its beneficial influence on the transition from AKI to CKD.

## Figures and Tables

**Table 1:** Primers used in this study for PCR (mouse).

Gene	Gene Accession Number	Forward	Reverse
*GAPDH*	NM_008084.4	5′-CATCACTGCCACCCAGAAGACTG-3′	5′-ATGCCAGTGAGCTTCCCGTTCAG-3′
*IL-*6	NM_031168.2	5′-TACCACTTCACAAGTCGGAGGC-3′	5′-CTGCAAGTGCATCATCGTTGTTC-3′
*TNF-α*	NM_013693.3	5′-GGTGCCTATGTCTCAGCCTCTT-3′	5′-GCCATAGAACTGATGAGAGGGAG-3′
*IL-1β*	NM_008361.4	5′-CCACAGACCTTCCAGGAGAATG-3′	5′-GTGCAGTTCAGTGATCGTACAGG-3′

## Data Availability

Data are contained within this article.
